# Supplementary figures and images for: The Emerging Role of IGF2BP2 in Cancer Therapy Resistance: From Molecular Mechanism to Future Potential
Source: Int J Mol Sci. 2024 Nov 12;25(22):12150. doi: 10.3390/ijms252212150 (PMC11595103; doi:10.3390/ijms252212150)

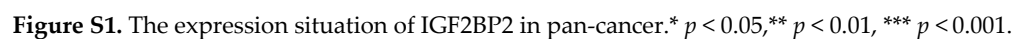

**Figure S1.** The expression situation of IGF2BP2 in pan-cancer.\*  $p < 0.05$ ,\*\*  $p < 0.01$ ,\*\*\*  $p < 0.001$ .

Supplement: Supplementary file 1 [file ijms-25-12150-s001.zip › ijms-3291504-supplementary.pdf]
